# Improving Ethnic Diversity in Cancer Trials Through Healthcare Interpreter Training

**DOI:** 10.1002/cam4.71071

**Published:** 2025-08-01

**Authors:** Suzanne J. Grant, Mayra Ouriques, Abhijit Pal, Sharon Lee, Sheetal Challam, Lindsey Jasicki

**Affiliations:** ^1^ NICM Health Research Institute, Western Sydney University Penrith South DC New South Wales Australia; ^2^ Cancer Institute NSW Sydney NSW Australia; ^3^ Liverpool Hospital and Bankstown Hospital– South Western Sydney Local Health District Sydney NSW Australia; ^4^ Western Sydney University Penrith South DC New South Wales Australia; ^5^ University of Sydney Faculty of Medicine and Health Sydney Australia; ^6^ Research & Education Network Western Sydney Local Health District Sydney NSW Australia

**Keywords:** clinical trials, culturally and linguistically diverse (CALD), education and training, equitable outcome, ethnic minorities, healthcare interpreters

## Abstract

**Introduction:**

People with cancer from culturally and linguistically diverse (CALD) backgrounds who are not proficient in English face many challenges in accessing clinical trials. Clinical trials offer opportunities to access cutting‐edge therapies for cancer management, with opportunities for longer survival and/or better quality of life. Inequitable access to these clinical trials not only reduces the validity of research findings, but also exacerbates the known disparities in cancer outcomes for these populations. Australia is a migrant majority country, with certain areas having large proportions of people who do not speak English—research has shown that this group has a lower rate of trial participation than those who can speak English. There is no available specific training in cancer clinical trials or research terminology for healthcare interpreters (HCIs). Research has shown that inadequately trained interpreters are a recognized barrier to clinical trial access for patients who are not proficient in English. This two‐phase quality improvement project, including a baseline knowledge survey and subsequent training modules, was undertaken to build workforce capacity for interpreters in cancer clinical trials.

**Methods:**

Phase 1: Subject matter experts and NSW Healthcare Interpreting Services managers codeveloped a survey to identify knowledge and skill gaps. HCIs across NSW (approx. 700) were invited to participate in a survey via an anonymous link (Qualtrics). Phase 2: Training was developed comprising five sections (basic concepts of clinical trials, governance and ethics, phases, informed consent and role of interpreters) using a blend of videos and presentations, interactive polls, and discussions. Pretraining and post‐training surveys were conducted to assess learnings. Statistical analysis used descriptive statistics and *t*‐tests.

**Results:**

In Phase 1, 133 interpreters responded to an initial online survey (response rate of 19%). The majority (71%) had been working as interpreters for more than 10 years. Clinical trial interpreting experience was limited; 34% had never interpreted for a clinical trial. Mean knowledge accuracy for clinical trial concepts was 68%, with uncertainty/lack of knowledge around randomization, clinical trial phases, and uncertainty around governance/ethics and clinical trial sponsors. In Phase 2, 92 interpreters attended in‐person or online training. Training increased mean accuracy in knowledge items about cancer clinical trials from 74% prior to training to 91% after the training. Confidence in understanding clinical trial terminology increased from 20% to 62% after training.

**Conclusion:**

Training for HCIs improved knowledge and confidence in understanding cancer clinical trial principles and terminology, building competency to provide better service to people from CALD backgrounds. The training modules developed will be made available online for statewide interpreter access. Future evaluation should track the impact on CALD trial participation to assess long‐term outcomes.

## Graphical abstract

In a survey of healthcare interpreters (HCI) about cancer clinical trials, key knowledge and skills gaps were identified. Training was developed and delivered for interpreters, improving knowledge and confidence in cancer clinical trials, building competency to provide improved interpreter service for culturally and linguistically diverse (CALD) patients who are considering participation in clinical trials.

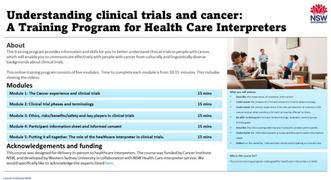

## 1 Introduction

People diagnosed with cancer are often overwhelmed and unsure about the treatment options available to them. The treating cancer clinician may recommend the option of participating in a clinical trial as part of their treatment—international consensus guidelines, such as those from the National Comprehensive Cancer Network (NCCN), recommend cancer clinical trial participation when a standard of care is not available for advanced cancers [1]. Clinical trials provide patients with access to new therapies that may not be available via standard of care [2]. Information provided to patients to consider participating in a clinical trial includes a patient information and consent form (PICF) that has been approved by a human research ethics committee. Information contained in PICF and other resources to support understanding and decision making can be technical and daunting for patients. These challenges are compounded for people with cancer whose preferred language is not English; it is well recognized in the literature that clinical trial PICF are complex and jargon heavy, and are challenging even for those who are proficient in English [3, 4].

Participation in clinical trials remains inequitable, with barriers that are multifaceted [5, 6]. Barriers to participation can affect individuals from rural and regional areas, implicit bias, lack of trust, attitudes and beliefs, and specific factors related to language and communication [7, 8, 9]. Poor health literacy and being unable to read or speak English are common barriers to clinical trial participation [9].

Research, including clinical trials, includes a sample size from the general population. The population sample size should reflect the diversity of the general population to support the transferability of analysis to the real world. Research that is not inclusive or reflective of our diverse communities impacts the external validity of research findings but also exacerbates the known disparities in cancer outcomes for these populations [10]. We have made substantial advances in cancer treatment in the last decade, including significant cure rates in previously lethal cancers, including immunotherapy for melanoma, which has resulted in 50% survival at 5 years [11] and biomarker targeted therapy in lung cancer, where patients can live for 7 years [12] and longer—these outstanding outcomes were realised through well conducted clinical trials and were available only to clinical trial participants for the first several years in their development.

Barriers to recruiting participants to a cancer clinical trial include inequitable inclusion criteria, access to cancer centers where clinical trials are conducted, competing demands such as work and caregiving responsibilities, and in addition for culturally and linguistically diverse (CALD) participants a lack of translated materials and/or availability of interpreters [10, 13]. There is also a lack of trust in research in healthcare from ethnic minorities [14, 15]. Studies report that when asked to participate, Black, Hispanic, and Asian patients had enrolment rates comparable to White Caucasian patients, indicating a differential in how racial and ethnically diverse groups are invited to participate [16, 17], or in some cases not invited to participate. Racial and ethnic minorities may be perceived as less promising participants [8].

In Australia, one in five people speak a language other than English at home, and in New South Wales, over half of the population (50.3%) have at least one parent born overseas. Language and limited English proficiency have been identified as a barrier to clinical trial participation [18]. Smith et al. analysed 19,453 patients in South West Sydney (a particularly diverse area comprising large populations of Arabic and Vietnamese patients) and demonstrated that trial participation was significantly lower in CALD than in non‐CALD patients (5.7% vs. 8.4%). They also found that CALD patients whose preferred language was not English were less likely to participate than non‐CALD (odds ratio [OR], 0.45) and CALD whose preferred language was English (OR, 0.53) [18].

Inadequately trained healthcare interpreters (HCIs), known as medical interpreters in the United States, have been identified as a barrier to clinical trial access for patients who are not proficient in English in Australia [19]. Professional HCIs undergo training in medical terminology, awareness of cultural differences, and competency in specialised medical settings. There is no available specific training in cancer clinical trials or research terminology for HCIs. Ensuring HCIs have the appropriate skillset and knowledge of medical terminology around cancer clinical trials will likely provide a better service to ethnic minorities, whose preferred language is not English, by opening up possible participation in clinical trials and strengthening the external validity of research outcomes. Engagement of HCIs, who are reflective of under‐represented populations, may also help address issues of trust. A sense of trust may assist in reducing attitudinal barriers such as the fear of being a “guinea pig,” which is so often perceived by minority groups [15, 17, 20].

In 2022, the Cancer Institute NSW, Australia, undertook a consultation process to improve awareness and involvement in cancer clinical trials in the CALD communities. The Cancer Institute NSW is a cancer control agency, established under the Cancer Institute NSW (2003) Act to lessen the impact of cancer across the State by working with the health system and community. The Cancer Institute NSW identified a potential facilitator to improving cultural diversity was to work with the healthcare interpreting services (HCIS) to enhance knowledge about cancer clinical trials. The NSW HCIS provides access to professional interpreting services in over 120 languages, including Auslan. The literature, to date, shows scant attention to training or education for interpreters regarding clinical trials, with only one initiative identified [21]. The aim of this paper is to report on a quality improvement project to provide NSW HCIS staff with training on cancer clinical trials. The goal was to increase workforce capability and improve the cultural diversity of participants in cancer clinical trials in NSW.

## 2 Materials and Methods

The project was conducted in two phases. In Phase 1, we conducted a cross‐sectional survey to investigate the baseline knowledge and training preferences of HCIs. In Phase 2, we developed a programme of training for HCIS. An assessment of understanding and confidence regarding clinical trials was undertaken before and after the delivery of the training developed.

### 2.1 Phase 1: HCIs Survey

#### 2.1.1 Survey Design

A group comprising subject matter experts in cancer clinical trials, HCIS Managers, and Cancer Institute NSW staff codesigned a survey (training needs assessment) to identify knowledge and skill gaps, preferred delivery styles and modes, and to provide direction for the training.

The initial survey tool was adapted from a project undertaken by the Massachusetts General Hospital Cancer Center (MGHCC) in collaboration with the Dana‐Farber/Harvard Cancer Center (DF/HCC) and the Cambridge Health Alliance to facilitate access to early phase clinical trial enrolment for people with limited English proficiency [21]. The survey was pilot tested with staff from the HCIS and Cancer Institute NSW; none of these staff participated in the final survey. Based on the pilot test's feedback, the survey was adjusted to the final version (see Data S1). The survey sections covered participant characteristics, past clinical trial experience, confidence and knowledge in the conduct of clinical trials, attitudes and beliefs about clinical trials, and training delivery preferences. Completion of the survey was voluntary and anonymous; consent was implied by completing the survey. To prevent multiple completions by a single user, a Qualtrics setting placed a cookie on the users' browser, which prevented the survey from being retaken. The survey is reported according to the Consensus‐Based Checklist for Reporting Survey Studies (CROSS) (see Data S2) [22].

#### 2.1.2 Respondent Selection

Purposive sampling. We distributed the survey to NSW HCIS staff supporting public hospitals (approx. 700 full‐time, part‐time, casual) via an email link and QR code (Qualtrics). The survey was kept open for two weeks between 28th July and 12th August 2022. No reminder emails were sent to staff for completion.

#### 2.1.3 Sample Size Calculation

For a population size of 700, a sample of 102 was calculated, allowing for a 9% margin of error, and a confidence level of 95% (calculated using Qualtrics sample size calculator).

#### 2.1.4 Survey Analysis

Statistical analysis was completed in RStudio by a biostatistician. The formula used for calculating the response rate was the number of responses returned divided by the number of email invitations sent out, multiplied by 100. Descriptive statistics for single variables and subgroup analysis were undertaken to determine any correlation between variables and language interpreted, and length of time as an interpreter. Missing completely at random (MCAR) data was handled by the removal of variables from the analysis. Nonresponse and selection errors may have occurred in this survey, although the knowledge assessment results in the initial survey were similar to the pretest knowledge assessments conducted in Phase 2.

### 2.2 Phase 2: Codesign and Delivery of Training Program

**FIGURE 1:**
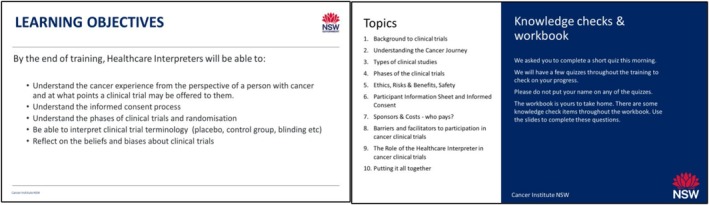
Training program: objectives and topics.

Training was developed using the results of the survey of interpreters at the NSW HCIS, through iterative discussions with HCIS managers, and consultation with key stakeholders to refine meaningful learning objectives (Figure 1). The final training was offered in a single session, and comprised five sections (basic concepts of clinical trials, governance and ethics, phases, informed consent and role of interpreters) using a blend of videos, interactive polls (online), and discussions.

**FIGURE 2:**
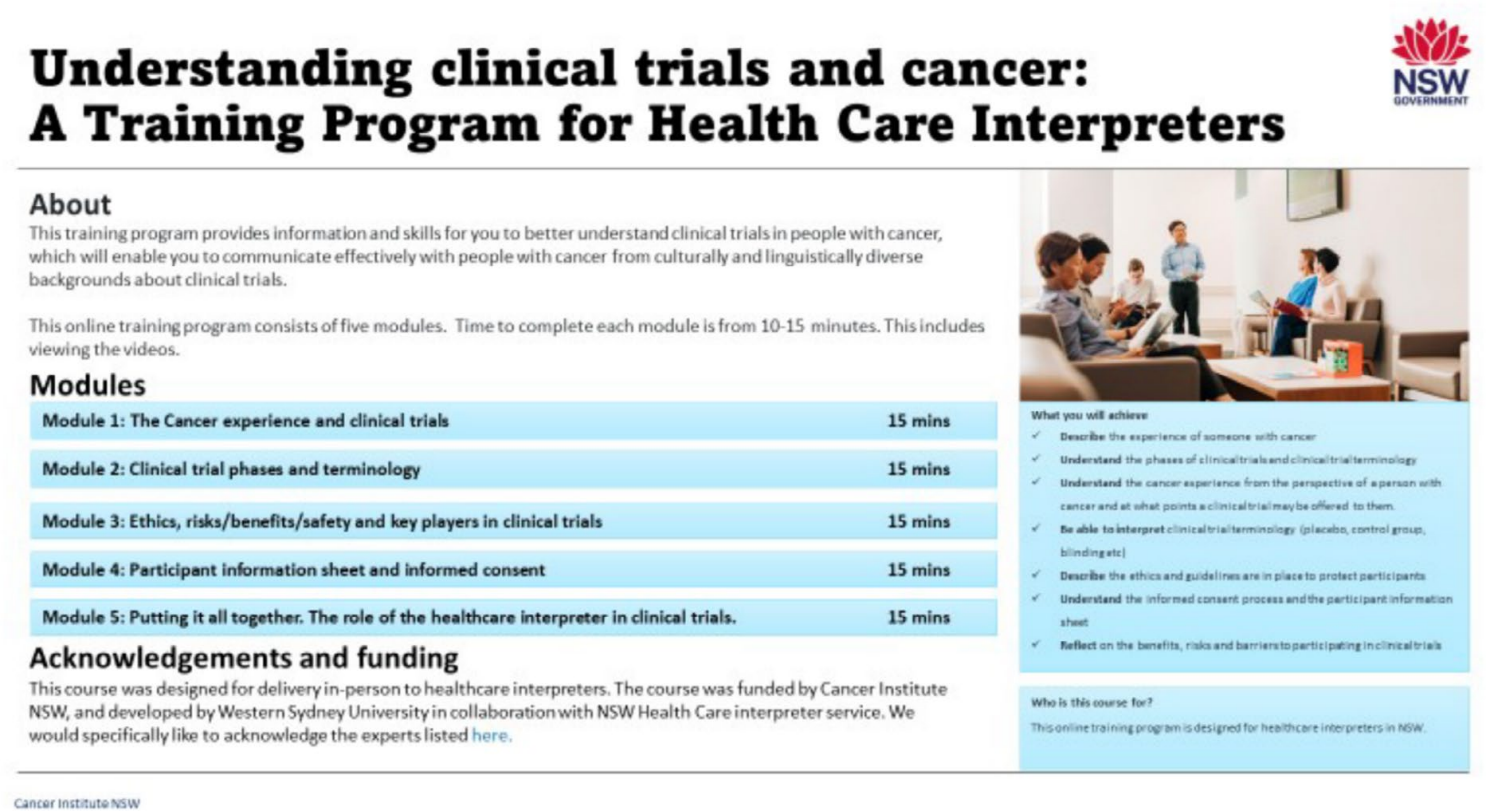
Training satisfaction.

Reflecting preferences from the survey and resource constraints, training was delivered both in‐person and live online during 2023. Three separate in‐person training sessions were conducted (4 h each), and the material was adapted for two live online sessions (2.5 h each) (Figure 2). Participants could choose to attend one of these training sessions.

Training was delivered by an experienced University educator with a Diploma in Education, a PhD and training in Good Clinical Practice (SG) and a Clinical Trials Coordinator with over 20 years experience (SL).

Participants were asked to complete a knowledge assessment prior to the training and at completion of the training session (pretest and post‐test). The assessments were identical apart from additional questions asked in the post‐test around satisfaction with the training (Data S3). The face‐to‐face assessments were administered paper‐based, and the online training assessments were distributed via a Qualtrics link to be completed directly before the training and straight after training.

As this was a quality improvement activity, ethical approval was not required.

## 3 Results

### 3.1 Phase 1: HCIs Survey

In Phase 1, 133 interpreters responded to the initial survey (response rate of 19%). The majority (71%) had been working as interpreters for > 10 years (Table 1). The top three languages that respondents interpreted were Arabic (*n* = 27), Mandarin (*n* = 25) and Cantonese (*n* = 18).

**TABLE 1:** Survey participant characteristics.

	*N* (%)
Years worked as an interpreter	
< 2 years	1 (1)
3–5 years	11 (8)
6–10 years	26 (20)
> 10 years	95 (71)
Experience in interpreting for clinical trials in the last year^a^	
Don't know/Don't remember	19 (16)
Never	52 (43)
Once	14 (12)
2–5 times	26 (21)
6–10 times	4 (3)
> 10 times	6 (5)
Languages interpreted	
Arabic	27 (19)
Mandarin	25 (18)
Cantonese	18 (13)
Korean	11 (8)
Spanish	6 (4)
Persian	5 (4)
French	4 (3)
Hindi	4 (3)
Vietnamese	4 (3)
Italian	4 (3)
Japanese	4 (3)
Dari	4 (3)
Assyrian	3 (2)
Punjabi	3 (2)
Hungarian	3 (2)
Serbian	3 (2)
Tamil	3 (2)
Other languages	11

^a^ Missing data *n* = 12.

Clinical trial interpreting experience was limited; 52 had not interpreted for a clinical trial in the previous year. Ten participants had interpreted for six or more clinical trials in the past year. Those respondents who had interpreted for more than six clinical trials were mainly interpreters of Arabic. Of the 27 interpreters of Arabic, 18 (67%) respondents had interpreted for at least one clinical trial, 12 had interpreted for more than one clinical trial in the previous year. For the 25 interpreters of Mandarin, 10 (40%) had interpreted for at least one clinical trial. For the 18 interpreters of Cantonese, seven (39%) had not interpreted for any clinical trials. The other language groups represented were too small for meaningful analysis.

Mean knowledge accuracy was 68%, with uncertainty/lack of knowledge around clinical trial concepts such as randomization, clinical trial phases, and uncertainty around governance, ethics, and clinical trial sponsors.

The majority of respondents with past clinical trial informed consent interpretation experience had been asked to sight translate clinical trial documents without prior preparation. This reportedly occurred frequently for 28% of respondents and occasionally for 47% of respondents. Most respondents only assisted in the clinical trial process during the initial consent (*n* = 49), while 20 respondents had been involved throughout the clinical trial.

**FIGURE 3:**
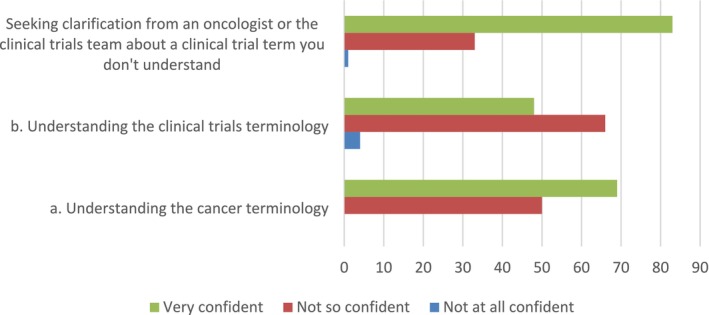
Online modules overview.

The majority of respondents were very confident understanding cancer terminology (58%) and were very confident in seeking clarification from an oncologist or the clinical trials team about a term they may not understand (71%). Respondents were not so confident in understanding the clinical trials terminology (59%) Figure 3.

In knowledge questions, respondents showed good knowledge around consent, placebo, and participant autonomy. There was uncertainty or a lack of knowledge around clinical trial concepts such as randomization (see Data S4 for details of responses). Uncertainty was also apparent around governance, ethics, and clinical trial sponsors. Clinical trial knowledge was consistent with perceived knowledge and confidence.

Training delivery preferences reflect the need for a mixed offering—online trainer‐led, face‐to‐face, and self‐directed (Data S4).

### 3.2 Phase 2: Training Delivery

In Phase 2, 92 HCIs attended in‐person (*n* = 52) or online training (*n* = 40). Details of participant characteristics for the online and in‐person training, and past clinical trial interpreting experience are presented in Data S5. Baseline characteristics of those attending the training were similar to those in the initial skills and knowledge gap survey. Nearly half of the participants had been interpreting for more than 10 years.

Table 2 shows the results of the initial (pretest) and final (post‐test) assessments. Following the training, participants' average accuracy on knowledge questions about cancer clinical trials increased from 74%–91%. Confidence in understanding clinical trial terminology rose from 20%–62%. This improvement was statistically significant, with the mean score increasing from 9.05 (SD 4.26) before training to 11.54 (SD 4.05) after training (*p* < 0.001). The pre‐ and post‐test quiz results demonstrated overall improvement. For instance, although participants' understanding of randomisation improved, as did the concept of why racial diversity in research matters, they remained areas needing targeted enhancement.

**TABLE 2:** Pretest and post‐test: knowledge^a^.

True/False questions	Pretest correct response	Post‐test correct response
	*n* = 65 (%)	*n* = 92 (%)
In a randomized clinical trial the doctor chooses the treatment the participant will receive. [FALSE]	17 (26)	58 (63)
A “Consent Form” list the potential benefits and risks when participating in a clinical trial. [TRUE]	49 (75)	81 (88)
A placebo is a look‐alike drug (pill) with no active ingredient. [TRUE]	48 (74)	80 (87)
People that agree to join a clinical trial have the right to withdraw from it at any time. [TRUE]	54 (83)	82 (89)
A Human Research and Ethics Committee is an independent committee that regulates and approves trials. {TRUE]	41 (63)	80 (87)
The “Consent Form” must be signed before a person can participate in a clinical trial. [TRUE]	56 (86)	82 (89)
In a randomized clinical trial, the treatment a person gets is decided by chance, like a flipping a coin. [TRUE]	28 (43)	72 (78)
If a clinical trial is about a very important clinical question, a doctor can force a patient to enter the trial. [FALSE]	53 (82)	81 (88)
A person is never put into a clinical trial without their knowledge [TRUE]	49 (75)	79 (86)
Those who participate in a clinical trial are helping others with cancer in the future [TRUE]	51 (78)	82 (89)
Once a person consents to join a trial and starts participating, they must remain in it until its end [FALSE]	45 (69)	77 (84)
A trial will be stopped if the investigators or the review board have concerns for the participants' safety [TRUE]	48 (74)	79 (86)
Standard of care is the treatment people will receive if not in the clinical trial [TRUE]	32 (49)	75 (82)
The racial diversity of clinical trials participants does not affect clinical trial result because medicine works the same for all people. [FALSE]	17 (26)	54 (59)

^a^ Numbers differed pretest to post‐test as participants arrived late at face to face and online training and were not able to complete the knowledge test.

**FIGURE 4:**
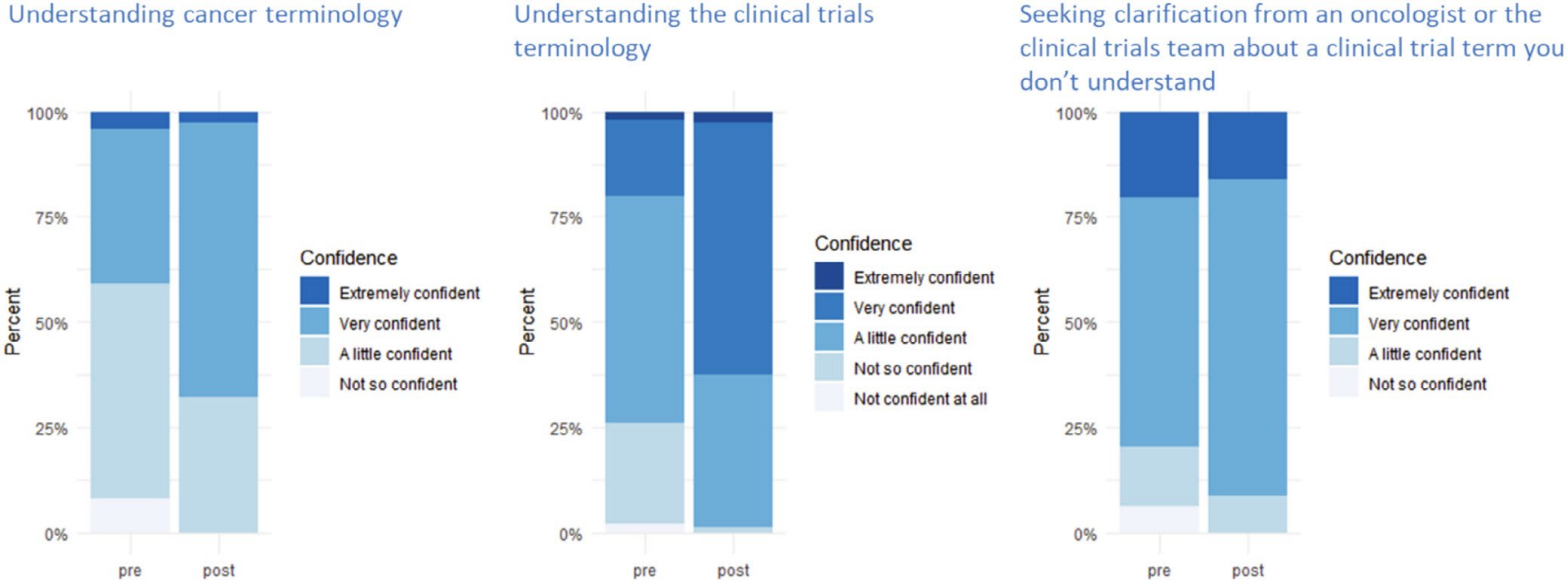
Confidence and perceived understanding. (responded = 119 missing = 14).

Confidence levels increased to being “very confident” in understanding clinical trial terminology from 20%–62% (Figure 4). Confidence in understanding cancer terminology also improved. Confidence in seeking clarification from an oncologist about a term that is not understood remained the same, although there were no longer any participants that selected ‘not so confident’ in the post‐test.

Separate questions were asked about ethics and informed consent. In response to the question “what is an ethics committee?” and “what is meant by informed consent?”, 78% and 89% (*p* < 0.067) responded correctly in the pretest and post‐test respectively to both questions.

### 3.3 Satisfaction With Training

**FIGURE 5:**
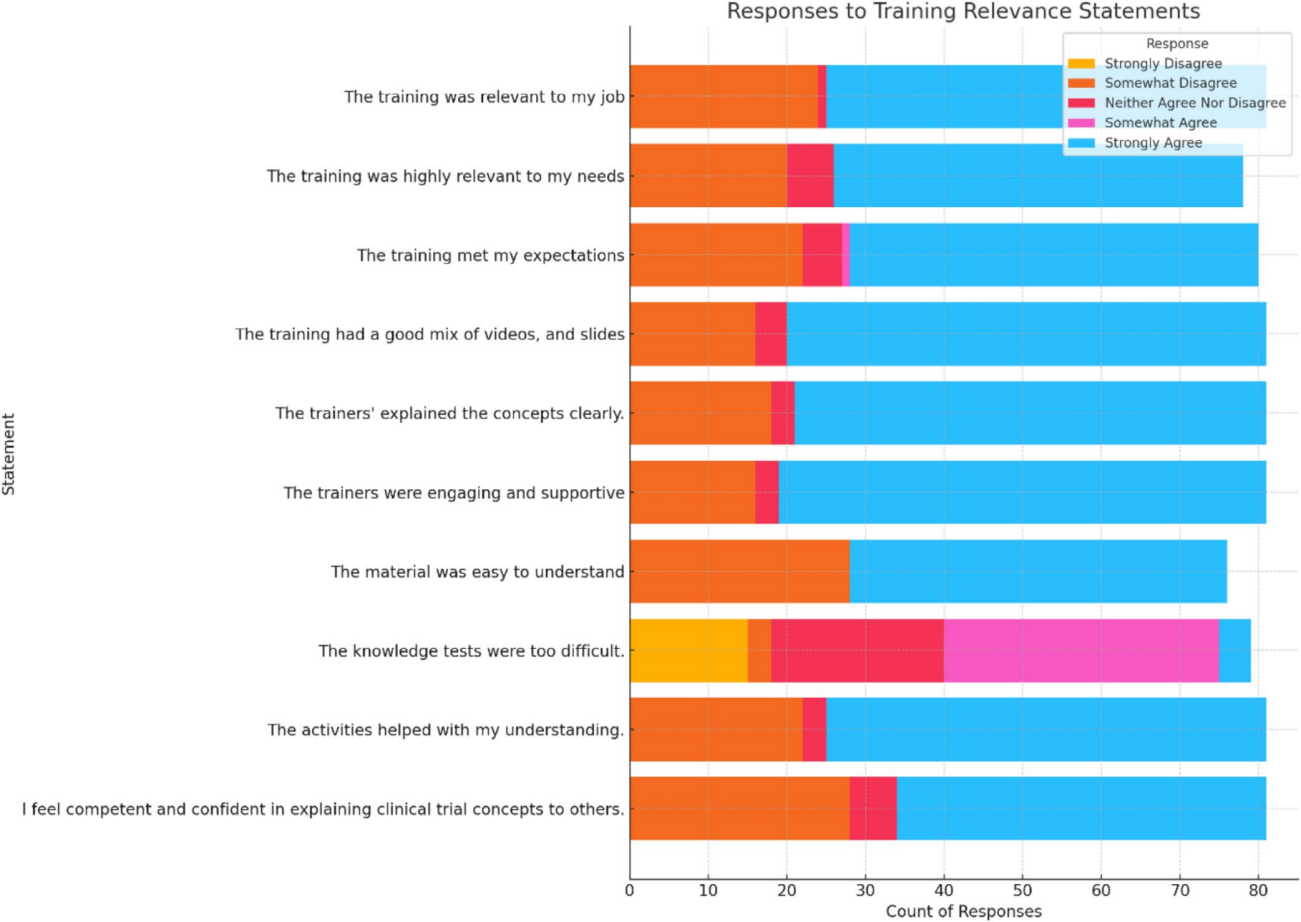
Confidence.

Participants in both the in‐person and online training variation expressed high levels of satisfaction. There were minimal responses indicating strong disagreement across almost all statements, demonstrating a generally favorable reception of the training's format and delivery. Figure 5 show that a substantial majority of participants strongly agreed that the training was relevant to their jobs, indicating high satisfaction with the content's applicability. For the statement “The training was highly relevant to my needs,” over half of the respondents also expressed strong agreement, suggesting that the training effectively addressed the specific needs of the participants.

Responses to the statement about the trainers being engaging and supportive were overwhelmingly positive, with the majority strongly agreeing. The distribution of responses varied more on statements regarding the balance of different training methods, such as the mix of videos and slides, suggesting mixed preferences among participants for these elements of the training programme.

## 4 Discussion

Training interpreters in cancer clinical trials was an innovative approach to address the inequitable and challenging issue of increasing cultural diversity and inclusion in Australian cancer clinical trials. To our knowledge, this is the first training of its kind to be conducted in Australia, and one of only a few initiatives globally [21].

Our initial survey revealed the expertise of interpreters in clinical trials was varied, with a small minority being actively and frequently engaged. Clear knowledge gaps were apparent in our initial workforce survey and baseline knowledge test, with uncertainty/lack of knowledge around clinical trial concepts and governance. Training increased mean accuracy in knowledge items about cancer clinical trials from 74%–91%. In two studies using a similar pretest–post‐test knowledge quiz, training led to significant increases in knowledge about cancer clinical trials [21, 23, 24].

Knowledge of clinical trial concepts prior to training indicated uncertainty around clinical trial concepts such as randomisation, clinical trial phases and governance, ethics committee review and the role of clinical trial sponsors. The lack of knowledge around “who is behind” a clinical trial echoes the most frequently reported barriers to participation in clinical trials by racial and ethnic minorities—mistrust of research, the medical system, sponsors and investigators [25]. Through the clarification of these roles and addressing misconceptions, we aimed to increase interpreters' confidence in communicating these concepts to potential participants and in the interpreters' interactions with clinical trial staff.

The training program addressed key gaps in confidence and understanding of clinical trial terminology, concepts such as randomisation, clinical trial governance, and participant eligibility. The positive findings concerning the effect of the training for interpreters on their confidence and knowledge were underpinned by very positive feedback and a high level of satisfaction from participants. Regarding the high level of satisfaction, it is important to note that participants did not have to pay for the training, which may have influenced response patterns.

The role of interpreters within a healthcare setting is complex, particularly in a cancer setting [26]. According to the Australian Institute of Interpreters and Translators (AUSIT), the main responsibility of interpreters is to accurately and impartially convey meaning between patients and healthcare providers, without adding, omitting, or altering the message. At the same time, interpreters often act as a “bridge between cultures” [26]. In Australia, patients who do not speak English are entitled to have information about clinical trials explained to them in their preferred language. Interpreters registered with the NSW Health Care Interpreter Service (HCIS) are trained in medical terminology and ethics, and they are expected to support patients during consent discussions. However, there is no specific training provided on clinical trials or research protocols. As a result, interpreters may translate complex research concepts—like randomisation or placebo—without formal exposure to them.

The training provided to interpreters as part of this project has since been expanded and adapted into a stand‐alone set of six online modules. These modules cover the same core content, with the addition of video materials and a role‐play involving an oncologist, interpreter, and patient. The modules will be freely accessible to interpreters at any time via the NSW Government's evidence‐based information platform eviQ [27].

This study has several limitations. Our sample may not be fully representative, as participants self‐selected into the training and survey, with only 19% responding initially. While interpreters came from diverse regions and language groups across NSW, the findings may not generalize to other settings. The training was developed in the context of oncology, so some content may not apply to clinical trials in other areas.

We reported group‐level quiz results rather than tracking individual progress, and while the questions were based on a needs assessment, some key concepts—like randomisation—may have been oversimplified. Although many interpreters said they felt confident asking clinicians for clarification, this assumes they recognized when they did not understand—a substantial assumption, especially for more complex concepts. Our project does not provide an indication of how often this confidence may translate into practice, given time pressures and other dynamics in real world settings.

We recognize that misunderstandings around concepts like randomisation may raise concerns, which could impact how information is conveyed to patients and the integrity of informed consent.

The training aimed to enhance communication about clinical trials, but its direct impact on increasing participation of CALD populations cannot be isolated from broader statewide and national initiatives to improve diversity in clinical trial participation.

Finally, we did not ask about participants' prior experience with cancer‐related interpreting, which likely influenced their baseline knowledge and learning needs. While the training was free of charge, there may have been indirect costs—such as unpaid time off—that affected who was able to attend.

We acknowledge that improving equity in cancer clinical trials in Australia is a complex task with barriers at site, sponsor, and community levels. In this context, equity refers to ensuring that all population groups, including those from CALD backgrounds, have opportunities to participate in clinical research. Progress toward equity can be measured through metrics such as invitation, enrolment rates, interpreter use, and participant satisfaction. Action is required at each level of governance. These include reducing clinician bias, improving cultural awareness about research among clinicians, improving trust between hospitals and ethnic communities. However, the interpreter is a key focal point in the journey and we believe that providing them with the knowledge base to understand the complex concepts around human research is important.

## 5 Conclusion

Including participants from diverse backgrounds in clinical trials is essential to understanding variations in biologic differences in drug effectiveness and ensuring results apply to all populations [28]. This study showed that interpreter training can improve understanding of cancer clinical trial concepts. Building interpreter competency is a practical step to reducing barriers to participation in clinical trials by people from CALD backgrounds. The next step is to evaluate its impact through practical metrics—such as trial invitations, enrolment rates, and interpreter use for clinical trials—to determine whether this approach improves equity in participation.

## Author Contributions

**Suzanne J. Grant:** conceptualization (lead), data curation (lead), formal analysis (lead), investigation (lead), methodology (lead), writing – original draft (lead), writing – review and editing (lead). **Mayra Ouriques:** conceptualization (equal), investigation (supporting), methodology (supporting), writing – review and editing (supporting). **Abhijit Pal:** writing – review and editing (equal). **Sharon Lee:** data curation (supporting), formal analysis (supporting), writing – review and editing (equal). **Sheetal Challam:** conceptualization (lead), funding acquisition (lead), investigation (equal), methodology (equal), project administration (lead), writing – review and editing (supporting). **Lindsey Jasicki:** formal analysis (supporting), project administration (equal), writing – review and editing (equal).

## Ethics Statement

The course evaluation study is classified as exempt under the Revised Common Rule; therefore, obtaining informed consent was not required by the ethics committee. Participants indicated their willingness to complete the survey by providing written assent.

## Conflicts of Interest

Nil (S.L., S.G.) A.P. has received consulting fees from Novotech; educational expenses from AstraZeneca, Cipla, and Janssen; conference support from Pfizer; and travel support from Merck Sharp and Dohme.

## Supporting information


Data S1.

Data S2.

Data S3.

Data S4.

Data S5.

## Data Availability

Survey data is available on request.

## References

[1] “Patient Resources,” https://www.nccn.org/patientresources/patient‐resources/resources‐for‐patients‐caregivers/clinical‐trials.

[2] V. Jenkins and L. Fallowfield , “Reasons for Accepting or Declining to Participate in Randomized Clinical Trials for Cancer Therapy,” British Journal of Cancer 82, no. 11 (2000): 1783–1788. 10839291 10.1054/bjoc.2000.1142 PMC2363224

[3] Q. Duong , S. J. Mandrekar , S. J. Winham , K. Cook , A. Jatoi , and J. G. le‐Rademacher , “Understanding Verbosity: Funding Source and the Length of Consent Forms for Cancer Clinical Trials,” Journal of Cancer Education 36 (2021): 1248–1252. 32385740 10.1007/s13187-020-01757-7 PMC7648720

[4] L. Malik and J. Cooper , “A Comparison of the Quality of Informed Consent for Phase I Oncology Trials Over a 30‐Year Period,” Cancer Chemotherapy and Pharmacology 82 (2018): 907–910. 30151620 10.1007/s00280-018-3673-x

[5] C. M. Aldrighetti , A. Niemierko , E. van Allen , H. Willers , and S. C. Kamran , “Racial and Ethnic Disparities Among Participants in Precision Oncology Clinical Studies,” JAMA Network Open 4, no. 11 (2021): e2133205. 34748007 10.1001/jamanetworkopen.2021.33205 PMC8576580

[6] N. Duma , J. Vera Aguilera , J. Paludo , et al., “Representation of Minorities and Women in Oncology Clinical Trials: Review of the Past 14 Years,” Journal of Oncology Practice 14, no. 1 (2018): e1–e10. 29099678 10.1200/JOP.2017.025288

[7] C. P. Williams , N. Senft Everson , N. Shelburne , and W. E. Norton , “Demographic and Health Behavior Factors Associated With Clinical Trial Invitation and Participation in the United States,” JAMA Network Open 4, no. 9 (2021): e2127792–e2127792. 34586365 10.1001/jamanetworkopen.2021.27792 PMC8482053

[8] S. J. Niranjan , M. Y. Martin , M. N. Fouad , et al., “Bias and Stereotyping Among Research and Clinical Professionals: Perspectives on Minority Recruitment for Oncology Clinical Trials,” Cancer 126, no. 9 (2020): 1958–1968. 32147815 10.1002/cncr.32755

[9] D. H. Bodicoat , A. C. Routen , A. Willis , et al., “Promoting Inclusion in Clinical Trials‐A Rapid Review of the Literature and Recommendations for Action,” Trials 22, no. 1 (2021): 880. 34863265 10.1186/s13063-021-05849-7 PMC8643184

[10] J. M. Loree , S. Anand , A. Dasari , et al., “Disparity of Race Reporting and Representation in Clinical Trials Leading to Cancer Drug Approvals From 2008 to 2018,” JAMA Oncology 5, no. 10 (2019): e191870. 31415071 10.1001/jamaoncol.2019.1870 PMC6696743

[11] J. Larkin , V. Chiarion‐Sileni , R. Gonzalez , et al., “Five‐Year Survival With Combined Nivolumab and Ipilimumab in Advanced Melanoma,” New England Journal of Medicine 381, no. 16 (2019): 1535–1546. 31562797 10.1056/NEJMoa1910836

[12] T. Mok , D. R. Camidge , S. M. Gadgeel , et al., “Updated Overall Survival and Final Progression‐Free Survival Data for Patients With Treatment‐Naive Advanced ALK‐Positive Non‐Small‐Cell Lung Cancer in the ALEX Study,” Annals of Oncology 31, no. 8 (2020): 1056–1064. 32418886 10.1016/j.annonc.2020.04.478

[13] J. M. Unger , R. Vaidya , D. L. Hershman , L. M. Minasian , and M. E. Fleury , “Systematic Review and Meta‐Analysis of the Magnitude of Structural, Clinical, and Physician and Patient Barriers to Cancer Clinical Trial Participation,” JNCI Journal of the National Cancer Institute 111, no. 3 (2019): 245–255. 30856272 10.1093/jnci/djy221 PMC6410951

[14] W. T. Hu , S. M. Bergren , D. K. Dychtwald , Y. Ma , and X. Q. Dong , “Variations in Racial and Ethnic Groups' Trust in Researchers Associated With Willingness to Participate in Research,” Humanities and Social Sciences Communications 10, no. 1 (2023): 466. 38650745 10.1057/s41599-023-01960-z PMC11034911

[15] K. Allison , D. Patel , and R. Kaur , “Assessing Multiple Factors Affecting Minority Participation in Clinical Trials: Development of the Clinical Trials Participation Barriers Survey,” Cureus 14, no. 4 (2022): e24424. 35637812 10.7759/cureus.24424 PMC9127181

[16] J. M. Unger , D. L. Hershman , C. Till , et al., ““When Offered to Participate”: A Systematic Review and Meta‐Analysis of Patient Agreement to Participate in Cancer Clinical Trials,” JNCI Journal of the National Cancer Institute 113, no. 3 (2021): 244–257. 33022716 10.1093/jnci/djaa155 PMC7936064

[17] B. Brijnath , R. Muoio , P. Feldman , et al., ““We Are Not Invited”: Australian Focus Group Results on How to Improve Ethnic Diversity in Trials,” Journal of Clinical Epidemiology 170 (2024): 111366. 38631530 10.1016/j.jclinepi.2024.111366

[18] A. B. Smith , M. Agar , G. Delaney , et al., “Lower Trial Participation by Culturally and Linguistically Diverse (CALD) Cancer Patients Is Largely due to Language Barriers,” Asia‐Pacific Journal of Clinical Oncology 14, no. 1 (2018): 52–60. 29083094 10.1111/ajco.12818

[19] A. Pal , B. Smith , C. Allan , D. Karikios , and F. Boyle , “Improving Access to Cancer Clinical Trials for Patients From Culturally and Linguistically Diverse Backgrounds in Australia: A Survey of Clinical and Research Professionals,” JCO Oncol Pract 19, no. 11 (2023): 1039–1047. 37677123 10.1200/OP.23.00291

[20] R. L. Comis , J. D. Miller , C. R. Aldigé , L. Krebs , and E. Stoval , “Public Attitudes Toward Participation in Cancer Clinical Trials,” Journal of Clinical Oncology 21, no. 5 (2003): 830–835. 12610181 10.1200/JCO.2003.02.105

[21] K. Donelan , K. Hobrecker , L. Schapira , J. R. Mailhot , B. H. Goulart , and B. A. Chabner , “Medical Interpreter Knowledge of Cancer and Cancer Clinical Trials,” Cancer 115, no. 14 (2009): 3283–3292. 19484791 10.1002/cncr.24377 PMC2785507

[22] A. Sharma , N. T. Minh Duc , T. Luu Lam Thang , et al., “A Consensus‐Based Checklist for Reporting of Survey Studies (CROSS),” Journal of General Internal Medicine 36, no. 10 (2021): 3179–3187. 33886027 10.1007/s11606-021-06737-1 PMC8481359

[23] R. K. Schutt , L. Schapira , J. Maniates , J. Santiccioli , S. Henlon , and J. A. Bigby , “Community Health Workers' Support for Cancer Clinical Trials: Description and Explanation,” Journal of Community Health 35, no. 4 (2010): 417–422. 20352478 10.1007/s10900-010-9267-0

[24] L. Schapira and R. Schutt , “Training Community Health Workers About Cancer Clinical Trials,” Journal of Immigrant and Minority Health 13, no. 5 (2011): 891–898. 21181445 10.1007/s10903-010-9432-7

[25] J. G. Ford , M. W. Howerton , G. Y. Lai , et al., “Barriers to Recruiting Underrepresented Populations to Cancer Clinical Trials: A Systematic Review,” Cancer 112, no. 2 (2008): 228–242. 18008363 10.1002/cncr.23157

[26] P. N. Butow , E. Lobb , M. Jefford , et al., “A Bridge Between Cultures: Interpreters' Perspectives of Consultations With Migrant Oncology Patients,” Supportive Care in Cancer 20, no. 2 (2012): 235–244. 21110046 10.1007/s00520-010-1046-z

[27] NSW, C.I , ”eviQ Cancer Treatments Online,” (2025), https://www.eviq.org.au/.

[28] K. Bibbins‐Domingo and A. Helman , Improving Representation in Clinical Trials and Research: Building Research Equity for Women and Underrepresented Groups, (National Academies Press, 2022). 36137057

