# Supplementary material for: Improving Ethnic Diversity in Cancer Trials Through Healthcare Interpreter Training
Source: Cancer Med. 2025 Aug 1;14(15):e71071. doi: 10.1002/cam4.71071 (PMC12314417; doi:10.1002/cam4.71071)
Supplement: Supplementary file 1 — Data S1. [file CAM4-14-e71071-s004.docx]

Healthcare Interpreting Service and Cancer Clinical Trials

Start of Block: CONSENT

Introduction The Cancer Institute NSW is asking for your contribution to this short survey. The purpose of the survey is to understand the training needs of an interpreter to support patients in participating in a clinical trial. A clinical trial is a type of research that studies a test or treatment given to people. When completing this survey, you are not required to provide your details. Your identity will remain anonymous. Your participation is voluntary and very valuable. This survey will take approximately 8 minutes.

- Yes I Consent (1)
- No I do not Consent (2)

End of Block: CONSENT

Start of Block: DEMOGRAPHICS

Q1 How long have you been working as a Healthcare Interpreter?

- 0 to 2 years (1)
- 3 to 5 years (2)
- 6 to 10 years (3)
- More than 10 years (4)

Q2 Which languages do you interpret?

- Mandarin (1)
- Arabic (2)
- Cantonese (3)
- Vietnamese (4)
- Italian (5)
- Greek (6)
- Tagalog/Filipino (7)
- Hindi (8)
- Spanish (9)
- Punjabi (10)
- German (11)
- Other........ (12) __________________________________________________

End of Block: DEMOGRAPHICS

Start of Block: Experience

Q3 How many clinical trials participants did you assist in the last year?

- 0 (1)
- 1 (2)
- 2-5 (3)
- 6-10 (4)
- More than 10 (5)
- I don’t know/ don’t remember. (6)

Q4 How confident are you in:

|  | Not at all confident (1) | Not so confident (2) | A little confident (3) | Very confident (4) | Extremely confident (5) |
| --- | --- | --- | --- | --- | --- |
| a. Understanding the cancer terminology (1) |  |  |  |  |  |
| b. Understanding the clinical trials terminology (2) |  |  |  |  |  |
| c. Seeking clarification from an oncologist or the clinical trials team about a clinical trial term you don’t understand (3) |  |  |  |  |  |

Q5 How often have you been asked to sight translate an English Consent document or treatment protocol to a client without prior preparation?

- Very often (1)
- Sometimes (2)
- Occasionally (3)
- Rarely (4)
- Never (5)
- Have never interpreted for a clinical trial (6)

Q6 If you have interpreted for a client going through a clinical trial, what was your experience? Select all that apply.

- Have not interpreted for a clinical trial (1)
- I received information about the trial before seeing the client. (2)
- The doctor or the clinical trials team explained the trial to me before seeing the client (3)
- I only assisted during the initial consent process. (4)
- I assisted during the clinical trial participation, consent and follow up visits (5)
- Other___________ (6) __________________________________________________

End of Block: Experience

Start of Block: CLINICAL TRIAL KNOWLEDGE

Q7 Please answer to the best of your knowledge.

|  | TRUE (1) | FALSE (2) | UNSURE (3) |
| --- | --- | --- | --- |
| a) In a randomised clinical trial the doctor chooses the treatment option to give the participant. (1) |  |  |  |
| b) A “Consent Form” outlines the potential benefits and risks of participating in a clinical trial. (2) |  |  |  |
| c) A placebo is a look-alike drug (pill) with no active ingredient (3) |  |  |  |
| d) People that agree to enter a clinical trial have the right to withdraw from it at any time. (4) |  |  |  |
| e) Human Research and Ethics Committee is an independent committee that regulates and approves trials. (5) |  |  |  |
| f) The “Consent Form” must be signed before a person can participate in a clinical trial. (6) |  |  |  |
| g) In a randomised clinical trial, the treatment a person gets is decided by chance. (7) |  |  |  |
| h) If a clinical trial is about a very important clinical question, a doctor can force a patient to enter the trial. (10) |  |  |  |
| i) Those who participate in a clinical trial are helping others with cancer in the future. (11) |  |  |  |
| j) Anyone with cancer is eligible to participate in a cancer clinical trial (12) |  |  |  |
| k) Once a person consents to join a trial and starts participating, they are forced to remain in it until the end (13) |  |  |  |
| l) A trial will be stopped if the investigators or review board have concerns for the participants' safety (15) |  |  |  |
| m) Standard of care is the treatment people will receive if not in the clinical trial (16) |  |  |  |
| n) Clinical trials are only sponsored and financially supported by drug companies. (17) |  |  |  |

End of Block: CLINICAL TRIAL KNOWLEDGE

Start of Block: BELIEFS

Q8 Please read the following statements that people have made about health care research. After each statement, click if you agree or disagree with the statement.

|  | AGREE (1) | DISAGREE (2) | DON'T KNOW (3) |
| --- | --- | --- | --- |
| A. Health care research benefits researchers, not the community. (1) |  |  |  |
| B. There should be more health care research occurring in minority communities. (2) |  |  |  |
| C. Participants’ rights are protected in health care research. (3) |  |  |  |
| D. If someone I love learned they were at high risk for developing cancer, I’d encourage him/her to participate in research studies about cancer treatments (4) |  |  |  |
| E. I believe that increasing awareness about clinical trials is important (5) |  |  |  |
| F. I believe that cancer research is important in finding better ways to prevent, diagnose and treat cancer (6) |  |  |  |

End of Block: BELIEFS

Start of Block: TRAINING PREFERENCES

Q9 If we, the Cancer Institute NSW, produced a resource to help healthcare interpreters understand more about cancer clinical trials, how would you like to receive it? Q9 Click on all those that apply.

- Self-directed training (online video) (1)
- Face to Face training (2)
- Self-directed training (pamphlets, flipcharts, brochures) (3)
- Trainer or teacher-led online training (4)
- I don't know (5)
- Other (6) __________________________________________________

End of Block: TRAINING PREFERENCES

Start of Block: Block 6

Q10 Please add any other comments here:

________________________________________________________________

________________________________________________________________

________________________________________________________________

________________________________________________________________

________________________________________________________________

End of Block: Block 6
