# Supplementary material for: Improving Ethnic Diversity in Cancer Trials Through Healthcare Interpreter Training
Source: Cancer Med. 2025 Aug 1;14(15):e71071. doi: 10.1002/cam4.71071 (PMC12314417; doi:10.1002/cam4.71071)
Supplement: Supplementary file 3 — Data S3. [file CAM4-14-e71071-s001.docx]

**Supplementary File 3: Health Care Interpreters Service Pretest and Posttest Assessment**

**Healthcare Interpreting Service Training**

# Cancer Clinical Trials

## Instructions

The Cancer Institute NSW is asking for your contribution to answer this survey. The purpose is to understand your knowledge about cancer clinical trials before the training session. We will collect a post-training survey as well. The result of this survey will assist the Cancer Institute NSW to improve the scope of the training. You are not required to provide your details.

Your identity will remain anonymous. Your participation is voluntary and very valuable.

**Q1** How long have you been working as a Healthcare Interpreter?

- 0-2 yrs
- 3 - 5 yrs
- 6 - 10 yrs
- More than 10 years

**Q2** Which languages do you interpret? Select language(s) you mainly interpret

Arabic

- Cantonese
- French
- German
- Greek
- Hindi
- Italian
- Korean
- Macedonian
- Mandarin
- Punjabi
- Spanish
- Tagalog/Filipino
- Vietnamese
- Other __________________________________________________

**Q4** How many clinical trials participants did you assist in the last year?

- 0
- 1
- 2-5
- 6-10
- More than 10
- I don’t know/ don’t remember.
- Not applicable. I did not assist any participants in a clinical trial.

**Q5** If you have interpreted for a patient going through a clinical trial, what was your experience? Select all that apply.

- Not applicable. I have not interpreted for a clinical trial
- I received information about the trial before seeing the client.
- The doctor or the clinical trials team explained the trial to me before seeing the client
- I only assisted during the initial consent process.
- I assisted during the clinical trial participation, consent and follow up visits
- Other (please specify) __________________________________________________

**Q6** What clinical trials are for? (Give two reasons)

- Reason 1 __________________________________________________
- Reason 2 __________________________________________________

| **Q7** Please answer to the best of your knowledge. | True | False | Unsure |
| --- | --- | --- | --- |
| In a randomised clinical trial the doctor chooses the treatment the participant will receive. |  |  |  |
| A “Consent Form” list the potential benefits and risks when participating in a clinical trial. |  |  |  |
| A placebo is a look-alike drug (pill) with no active ingredient |  |  |  |
| People that agree to join a clinical trial have the right to withdraw from it at any time. |  |  |  |
| Human Research and Ethics Committee is an independent committee that regulates and approves trials. |  |  |  |
| The “Consent Form” must be signed before a person can participate in a clinical trial. |  |  |  |
| In a randomised clinical trial, the treatment a person gets is decided by chance, like a flipping a coin. |  |  |  |
| If a clinical trial is about a very important clinical question, a doctor can force a patient to enter the trial. |  |  |  |
| A person is never put into a clinical trial without their knowledge |  |  |  |
| Those who participate in a clinical trial are helping others with cancer in the future. |  |  |  |
| Once a person consents to join a trial and starts participating, they are must remain in it until its end |  |  |  |
| A trial will be stopped if the investigators or the review board have concerns for the participants' safety |  |  |  |
| Standard of care is the treatment people will receive if not in the clinical trial |  |  |  |
| The racial diversity of clinical trials participants does not affect clinical trial result because medicine works the same for all people. |  |  |  |

**Q8** Who finances a clinical trial? **(Select all that apply)**

- Human Research and Ethics Committee
- Study Investigator (Doctor or Clinician)
- Government
- Universities or not-for-profit organisationss
- Drug or device company

**Q9** Which of the following is/are true about a placebo? **(Select all that apply.)**

- A. It has no active ingredient
- B. It is a lookalike drug.
- C. It allows blinding.
- D. It is used in all clinical trials.
- E. Is compared to the trial intervention to see which one works better

**Q10** In what phase of clinical trials is a new drug given to a small number of people, who are extensively tested to look at side effects and toxicities? (Select one only)

- A. Phase 1
- B. Phase II
- C. Phase III
- D. Phase IV

**Q11** What is an Ethics Committee? **(select one answer only)**

- A. People who like to talk a lot (A group of experienced people who understand research but with no authority to decide if a clinical trial can be conducted.)
- B. A group of people who think about hypothetical research
- C. A group of experienced people who will consider a research proposal and the degree to which ethical issues have been considered appropriately
- D. A group of experienced people who are useful to draw on when writing a research proposal

**Q12** What is meant by ‘informed consent’? (**Select one answer only.)**

- A. Telling people who you are and what you are studying
- B. The ethics committee is informed about the research
- C. Participants are given a clear indication what the research is about and what their involvement would entail
- D. Participants are informed about the findings of the research at the end of the project

**Q13** Who signs the informed consent document? **(select all that apply)**

- A. The participant
- B. A family member
- C. A witness
- D. The healthcare interpreter
- E. The research investigator
- F. The participant’s GP
- G. All of the above

**Q14** Which is the following is NOT a basic element of informed consent? **(select one)**

- A. Description of the study procedures (such as the number and type of blood tests, hospital visits etc)
- B. Voluntary participation
- C. Contact details of the Ethics Committee
- D. Number of participants in the clinical trial

**Q15** Informed consent in a clinical trial involves which of the following? **(select all that apply)**

- Agreeing in writing to be part of the clinical trial
- Being told about the risks of the clinical trial
- Being told about new information about the study drug or procedure after the clinical trial has started
- Reading and understanding the participant information sheet
- All of the above

**Q16** List three things that are usually included in Participant Information Sheet?

- 1. __________________________________________________
- 2. __________________________________________________
- 3. __________________________________________________

**Q17** How confident are you in:

|  | Not confident at all | Not so confident | A little confident | Very confident | Extremely confident |
| --- | --- | --- | --- | --- | --- |
| a. Understanding the cancer terminology |  |  |  |  |  |
| b. Understanding the clinical trials terminology |  |  |  |  |  |
| c. Seeking clarification from an oncologist or the clinical trials team about a clinical trial term you don’t understand |  |  |  |  |  |

**Q20** **Evaluation.**
This training is very new. Please take some time to provide feedback on the training. content, delivery, structure and trainer so that we can improve this course.

|  | Excellent | Good | Fair | Poor |
| --- | --- | --- | --- | --- |
| Overall, how would you rate the training today |  |  |  |  |

**Q21**

|  | Strongly agree | Somewhat agree | Neither agree nor disagree | Somewhat disagree | Strongly disagree |
| --- | --- | --- | --- | --- | --- |
| The training was relevant to my job |  |  |  |  |  |
| The material was easy to understand |  |  |  |  |  |
| The training had a good mix of videos, and slides |  |  |  |  |  |
| The training met my expectations |  |  |  |  |  |
| The trainers were engaging and supportive |  |  |  |  |  |
| I feel competent and confident in explaining clinical trial concepts to others. |  |  |  |  |  |
| The trainers' explained the concepts clearly. |  |  |  |  |  |
| The activities helped with my understanding. |  |  |  |  |  |
| The knowledge tests were too difficult. |  |  |  |  |  |
| The training was highly relevant to my needs. |  |  |  |  |  |

Q22 What did you like most about the training?

________________________________________________________________

Q23 What did you like least about the training?

________________________________________________________________

Q24 Did you think the content in the training material was sufficient? What could be added or improved?

______________________________________________________________________________________
