# Supplementary material for: Improving Ethnic Diversity in Cancer Trials Through Healthcare Interpreter Training
Source: Cancer Med. 2025 Aug 1;14(15):e71071. doi: 10.1002/cam4.71071 (PMC12314417; doi:10.1002/cam4.71071)
Supplement: Supplementary file 4 — Data S4. [file CAM4-14-e71071-s003.docx]

**Supplemental File 4: Results of Initial Survey**

**Clinical Trial Knowledge**

Respondents showed good knowledge around consent, placebo and participant autonomy. There was uncertainty or a lack of knowledge around clinical trial concepts such as randomization (Q1, Q7). Uncertainty was also apparent around governance around ethics and clinical trial sponsors. Clinical trial knowledge was consistent with perceived knowledge and confidence.

| **Table 1: Clinical trial knowledge** (Answered = 110 Skipped = 23) | True | False | Unsure |
| --- | --- | --- | --- |
| 1. In a randomised clinical trial the doctor chooses the treatment option to give the participant. | 42 | 37 | 30 |
| 1. A “Consent Form” outlines the potential benefits and risks of participating in a clinical trial.) | 96 | 5 | 8 |
| 1. A placebo is a look-alike drug (pill) with no active ingredient) | 82 | 7 | 19 |
| 1. People that agree to enter a clinical trial have the right to withdraw from it at any time. | 94 | 3 | 11 |
| 1. Human Research and Ethics Committee is an independent committee that regulates and approves trials. | 67 | 3 | 38 |
| 1. The “Consent Form” must be signed before a person can participate in a clinical trial. | 104 | 2 | 2 |
| 1. In a randomised clinical trial, the treatment a person gets is decided by chance. | 44 | 32 | 33 |
| 1. If a clinical trial is about a very important clinical question, a doctor can force a patient to enter the trial. | 1 | 102 | 6 |
| 1. Those who participate in a clinical trial are helping others with cancer in the future.) | 103 |  | 6 |
| 1. Anyone with cancer is eligible to participate in a cancer clinical trial | 27 | 54 | 28 |
| 1. Once a person consents to join a trial and starts participating, they are forced to remain in it until the end | 1 | 97 | 11 |
| 1. A trial will be stopped if the investigators or review board have concerns for the participants' safety | 102 | 0 | 7 |
| 1. Standard of care is the treatment people will receive if not in the clinical trial | 62 | 15 | 31 |
| 1. Clinical trials are only sponsored and financially supported by drug companies.) | 14 | 50 | 45 |

**Attitudes and Beliefs**

Attitudes and beliefs around clinical trials were generally supportive. The question about more health research in minority communities was not cancer specific.

| **Table 2: Attitudes & Beliefs** (Answered n=109, skipped 24) | Agree | Disagree | Don’t know |
| --- | --- | --- | --- |
| Health care research benefits researchers, not the community. | 6 | 99 | 4 |
| There should be more health care research occurring in minority communities | 82 | 10 | 16 |
| Participants’ rights are protected in health care research. | 100 | 2 | 7 |
| If someone I love learned they had were at high risk for developing cancer, I’d encourage him/her to participate in research studies about cancer treatments | 67 | 14 | 28 |
| I believe that increasing awareness about clinical trials is important | 102 | 1 | 5 |
| I believe that cancer research is important in finding better ways to prevent, diagnose and treat cancer | 106 | 1 | 2 |

**Training preferences**

The preferences for training were very mixed between self-directed (n=67) and face-to-face (n=67) or teacher led (n=61).

**Table 3: Training Preferences** (Answered = 108 Skipped = 25)

| Training delivery preference (respondents could select more than one) | n |
| --- | --- |
| Self-directed training (online video) | 67 |
| Face to Face training | 62 |
| Self-directed training (pamphlets, flipcharts, brochures) | 44 |
| Trainer or teacher-led online training | 61 |
| I don't know | 2 |
| Other | 3 |
