# Supplementary material for: Improving Ethnic Diversity in Cancer Trials Through Healthcare Interpreter Training
Source: Cancer Med. 2025 Aug 1;14(15):e71071. doi: 10.1002/cam4.71071 (PMC12314417; doi:10.1002/cam4.71071)
Supplement: Supplementary file 5 — Data S5. [file CAM4-14-e71071-s005.docx]

**Supplemental File 5: Initial Survey Participant Characteristics**

**Table 1. Survey Participant Characteristics**

| **Characteristic** | Pretest | Posttest |
| --- | --- | --- |
|  | N=65^1^ (%) | N=92 (%) |
| **Years worked as an interpreter** |  |  |
| < 2 years | 6 (9) | 3 (3) |
| 3-5 years | 10 (15) | 11 (12) |
| 6-10 years | 19 (29) | 26 (28) |
| >10 years | 30 (46) | 52 (56) |
| **Experience in interpreting for clinical trials in the last year^2^** |  |  |
| Don’t know/Don’t remember | 8 (14) | 16 (19) |
| Never | 29 (49) | 34 (40) |
| Once | 8 (14) | 13 (15) |
| 2-5 times | 13 (22) | 20 (24) |
| 6-10 times | 1 (2) | 1 (1) |
| >10 times | 0 | 0 |
| **Languages interpreted^3^** |  |  |
| Arabic | 15 (24) | 21 (23) |
| Mandarin | 11 (17) | 20 (22) |
| Cantonese | 3 (5) | 8 (9) |
| Korean | 2 (3) | 2 (2) |
| Spanish | 3 (5) | 6 (7) |
| Persian | 1 (2) | 1 (1) |
| Croation | 1 (2) | 2 (2) |
| Hindi | 0 | 1 (1) |
| Vietnamese | 4 (6) | 6 (7) |
| Italian | 3 (5) | 3 (3) |
| Japanese | 1 (2) | 1 (1) |
| Dari | 1 (2) | 1 (1) |
| Assyrian | 2 (3) | 1 (1) |
| Punjabi | 0 | 1 (1) |
| Thai | 1 (2) | 1 (1) |
| Serbian | 3 (5) | 4 (4) |
| Farsi | 1 (2) | 1 (1) |
| Macedonian | 1 (2) | 4 (4) |
| Russian | 2 (3) | 4 (4) |
| Chaldean | 1 (2) | 1 (1) |
| Bengali | 1 (2) | 2 (2.2) |

Notes: ^1^Participants arrived late at the in-person and online training and did not complete the pretest survey (n=27). ^2^Missing data pretest n=6; missing data posttest n=7 ^3^Participants may interpret for more than one language.

**Table 2: Past Clinical trial experience**

| **If you have interpreted for a patient going through a clinical trial, what was your experience? Select all that apply.** | **(n=52)** | **%** | **(n=78)** | **%** |
| --- | --- | --- | --- | --- |
| Not applicable. I have not interpreted for a clinical trial | 20 | 38.5% | 26 | 33.3% |
| I received information about the trial before seeing the client. | 1 | 1.9% | 7 | 9.0% |
| The doctor or the clinical trials team explained the trial to me before seeing the client | 0 | 0.0% | 10 | 12.8% |
| I only assisted during the initial consent process. | 22 | 42.3% | 33 | 42.3% |
| I assisted during the clinical trial participation, consent and follow up visits | 6 | 11.5% | 14 | 17.9% |
